# The Cellular Prion Protein Prevents Copper-Induced Inhibition of P2X_4_ Receptors

**DOI:** 10.4061/2011/706576

**Published:** 2011-10-19

**Authors:** Ramón A. Lorca, Lorena Varela-Nallar, Nibaldo C. Inestrosa, J. Pablo Huidobro-Toro

**Affiliations:** ^1^Departamento de Fisiología, Centro de Envejecimiento y Regeneración (CARE), Facultad de Ciencias Biológicas, P. Universidad Católica de Chile, Santiago 8331150, Chile; ^2^Departamento de Biología Celular y Molecular, Centro de Envejecimiento y Regeneración (CARE), Facultad de Ciencias Biológicas, P. Universidad Católica de Chile, Santiago 8331150, Chile

## Abstract

Although the physiological function of the cellular prion protein (PrP^C^) remains unknown, several evidences support the notion of its role in copper homeostasis. PrP^C^ binds Cu^2+^ through a domain composed by four to five repeats of eight amino acids. Previously, we have shown that the perfusion of this domain prevents and reverses the inhibition by Cu^2+^ of the adenosine triphosphate (ATP)-evoked currents in the P2X_4_ receptor subtype, highlighting a modulatory role for PrP^C^ in synaptic transmission through regulation of Cu^2+^ levels. Here, we study the effect of full-length PrP^C^ in Cu^2+^ inhibition of P2X_4_ receptor when both are coexpressed. PrP^C^ expression does not significantly change the ATP concentration-response curve in oocytes expressing P2X_4_ receptors. However, the presence of PrP^C^ reduces the inhibition by Cu^2+^ of the ATP-elicited currents in these oocytes, confirming our previous observations with the Cu^2+^ binding domain. Thus, our observations suggest a role for PrP^C^ in modulating synaptic activity through binding of extracellular Cu^2+^.

## 1. Introduction

Prion diseases are a group of fatal neurodegenerative disorders that are sporadic, inherited, or transmissible [1]. These include kuru and Creutzfeldt-Jakob disease in humans, scrapie in sheep and bovine spongiform encephalopathy in cattle. These pathologies are caused by the conformational transition of the native and predominantly *α*-helical cellular prion protein (PrP^C^) into a significantly more *β*-sheet-containing pathogenic isoform (PrP^Sc^) [2], which unlike PrP^C^, is insoluble in mild detergents and partially resistant to digestion with proteinase K [3]. PrP^C^ is a cell surface glycosylphosphatidylinositol-anchored protein that is mainly expressed in neurons and glial cells and to a lesser extent in several peripheral tissues [4, 5]. The normal physiological function of PrP^C^ remains elusive, although it has been related to signaling, neuroprotection, neuritogenesis, synaptic transmission, oxidative stress, and copper metabolism [6–11]. 

PrP^C^ binds copper ions with low micromolar affinity via histidine and glycine-containing peptide repeats in its N-terminal region [12–17]. This Cu^2+^ binding domain is located between residues 60–91 and consists of four identical repeats of the peptide sequence Pro-His-Gly-Gly-Gly-Trp-Gly-Gln. Although the number of octapeptide repeats varies in different species, in mammals this region is one of the most highly conserved [18] and therefore, very likely defines a functional domain of PrP^C^. *In vitro*, the octarepeat region has the capacity to reduce Cu(II) to Cu(I) [19, 20]. In addition, there is another Cu^2+^ binding site outside the octarepeat region [21–24] of higher affinity, in the order of nanomolar, that involves His96 and His111 [24]. PrP^C^ is localized presynaptically at central synapses [25–27] and is found in synaptic membranes and in synaptic vesicles [9, 28]. Furthermore, PrP^C^-null mice show an impaired long-term potentiation, suggesting that PrP^C^ is involved in normal synaptic function [10], and moreover, it has been shown that PrP^C^ is involved in regulating the presynaptic Cu^2+^ concentration and synaptic transmission [9].

The P2X family of nucleotide receptors forms non-selective cationic channels activated by extracellular adenosine triphosphate (ATP) [29]. These receptors are widely expressed in the central nervous system (CNS) [30–32] and are involved in synaptic transmission and plasticity including long-term potentiation as recently shown by us [33]. Interestingly, trace metals modulate P2X receptors, particularly, the P2X_4_ receptor subtype is differentially modulated by trace metals at physiological concentrations [34–37]. While Zn^2+^ facilitates the ATP-evoked currents, Cu^2+^ inhibits it in a concentration-dependent manner [37]. Previously, we demonstrated that the N-terminal octarepeat fragment of the PrP^C^ prevents and reverses the inhibitory action of Cu^2+^ on the P2X_4_ receptor when added to the media [38]. Herein, in an attempt to determine whether the PrP^C^-Cu^2+^ interaction is relevant to synaptic activity, we extended our investigations to test whether the full-length PrP^C^co-expressed with the P2X_4_ receptor may modulate *in situ* the Cu^2+^-induced inhibition of the ATP current gated by the P2X_4_ receptor.

## 2. Materials and Methods

### 2.1. Drugs and Chemicals

Copper chloride, ATP (as the tetrasodium salt), collagenase IA, and penicillin-streptomycin were purchased from Sigma Chemical Co (St Louis, Mo). All the salts used to prepare the Barth's incubation media and the recording solutions were analytically graded and were purchased from Merck (Darmstadt, Germany).

### 2.2. Oocyte Preparation, Injection, and Electrophysiological Recordings

A segment of the *Xenopus laevis* ovary lobe was surgically removed from adult anesthetized frogs; stages V-VI oocytes were manually defolliculated and then incubated with collagenase IA (1 mg/mL) for 30 min. Oocytes were manually injected with 7.5–12.5 ng cDNA coding for the rat P2X_4_ receptor with or without cDNA coding for the hamster prion protein (PrP-3F4), both cDNAs in plasmid pcDNA3, at 250 ng/*μ*L. After 48–72 h of incubation at 15°C in Barth's solution (in mM): 88 NaCl, 1 KCl, 2.4 NaHCO_3_, 10 HEPES, 0.82 MgSO_4_, 0.33 Ca(NO_3_)_2_, pH 7.5, supplemented with 10 IU/L penicillin/10 mg streptomycin, oocytes were clamped at −70 mV using the two-electrode voltage clamp technique with an OC-725C oocyte clamper (Warner Instrument Corp, Hamden, CT). ATP and CuCl_2_, dissolved in Barth's solution, were superfused at 2 ml/min. ATP-evoked currents were recorded with a 10 s ATP exposure applied regularly at 10–15 min intervals. These intervals were increased up to 25 min for maximal ATP concentrations in concentration-response curves protocols to decrease desensitization. Copper was applied for 30 s prior 10 *μ*M ATP (coapplied with CuCl_2_).

### 2.3. Confocal Microscopy

To study the distribution of PrP, oocytes were coinjected with the cDNA coding for the rat P2X_4_ receptor with the cDNA coding for mouse PrP-GFP (MmPrP-EGFP[25-266]-cDNA3). Oocytes, where P2X_4_ receptor expression was validated electrophysiologically, were directly analyzed on a Zeiss LSM 5 Pascal confocal microscope.

### 2.4. Western Blotting

After electrophysiological protocols, each oocyte injected with cDNa coding for P2X_4_ and PrP-3F4 was homogenized for 30 min in ice, using 40 *μ*L of lysis buffer per oocyte (100 mM NaCl, 20 mM Tris-HCl pH 7.4, 1% Triton X-100) supplemented with a protease inhibitors cocktail [39]. The extracts were centrifuged for 30 s at 14000 r.p.m. at 4°C and the supernatant was removed and resolved by 12% SDS-PAGE and transferred to nitrocellulose. Nonspecific binding sites were blocked with 5% (w/v) milk in Tris-Buffered Saline (TBS) 0.1% Tween (TBST) for 1 h. After blocking, blots were incubated with monoclonal anti-3F4 antibody [40], diluted 1 : 5000 in 3% (w/v) milk in TBST for 1 h at room temperature, followed by three 15 min washes in TBST at room temperature. The reactions were followed by incubation with anti-mouse antibody peroxidase labeled (Pierce, Rockford, IL) and developed by enhanced chemiluminescence.

### 2.5. Data Analysis

The average reduction of the ATP-gated current was normalized. The ATP and Cu^2+^ concentration-response curves were fitted to a sigmoid function using the GraphPad Prism software (San Diego, Cal). The median effective (EC_50_) or median inhibitory concentrations (IC_50_) for ATP or copper, respectively, were interpolated from these curves. Each protocol was performed in separate oocytes coming from at least two separate batches of oocytes. Mann-Whitney nonparametric Student's *t*-test was used for statistical analysis. A *P*  value < 0.05 was considered significant.

## 3. Results

### 3.1. The Expression of PrP-3F4 Did Not Change the ATP Concentration-Response Curve of P2X_4_ Receptors

**Figure 1:**
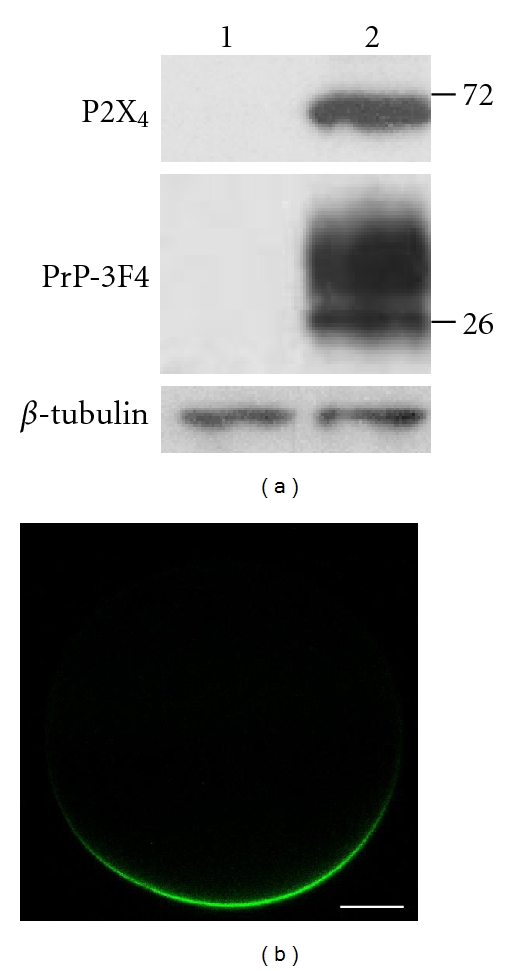
Coexpression of P2X_4_ and PrP^C^ in *X. laevis* oocytes. (a) Western blot of total lysate fractions from a non-injected oocyte (*left lane*, 1) and from an oocyte co-expressing P2X_4_ receptor and PrP-3F4 (*right lane*, 2). Numbers on the right are molecular weights in kDa. (b) Fluorescence microscopy of an oocyte co-expressing P2X_4_ receptor and PrP-GFP (green), bar = 10 *μ*M.

To evaluate whether the expression of PrP^C^ modulates the inhibition of the P2X_4_ receptor by Cu^2+^, we first evaluated the expression of PrP^C^ in oocytes co-injected with the cDNA coding for the hamster prion protein (PrP-3F4) and the cDNA coding for the rat P2X_4_ receptor.  Figure 1(a) shows the detection by western blot of P2X_4_ receptor and PrP-3F4 using an antibody that recognizes the 3F4 epitope [40]. *β*-Tubulin detection was used as a loading control. As observed, both proteins are strongly detected in an injected oocyte and not in the control noninjected oocyte. Then we analyzed the distribution of PrP^C^ in oocytes co-injected with the cDNA coding for the rat P2X_4_ receptor and the cDNA coding for PrP-GFP. Oocytes in which the expression of P2X_4_ receptor was verified electrophysiologically were analyzed in a confocal microscope to study the localization of PrP-GFP. As observed in Figure 1(b), PrP-GFP is located on the surface of injected oocytes. 

**Figure 2:**
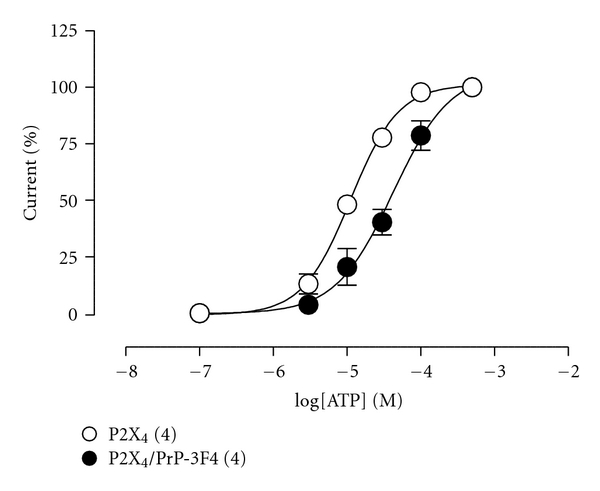
ATP concentration-response curves from oocytes expressing P2X_4_ receptor (open circles) or co-expressing P2X_4_ receptor and PrP-3F4 (closed circles). Symbols are mean values ± SEM, numbers in parenthesis are number of oocytes.

Then, we evaluated the ATP concentration-response curves in oocytes expressing the P2X_4_ receptor and coexpressing the P2X_4_ receptor and PrP-3F4. The presence of PrP-3F4 caused a slight, but not significant, reduction in the potency of ATP, reflected as an increase in its EC_50_ from 11.2 ± 1.1 *μ*M for P2X_4_ alone to 45.2 ± 9.4 *μ*M for P2X_4_/PrP-3F4 (*n* = 4, *P* = 0.0571, Figure 2), this slight displacement of ATP concentration-response curve in the presence of PrP-3F4 could represent a minor regulation of PrP-3F4 on P2X_4_ receptor activity.

### 3.2. The Co-Expression of P2X_4_ Receptors and PrP-3F4 Partially Prevents the Copper-Induced Inhibition of the ATP-Evoked Currents

**Figure 3:**
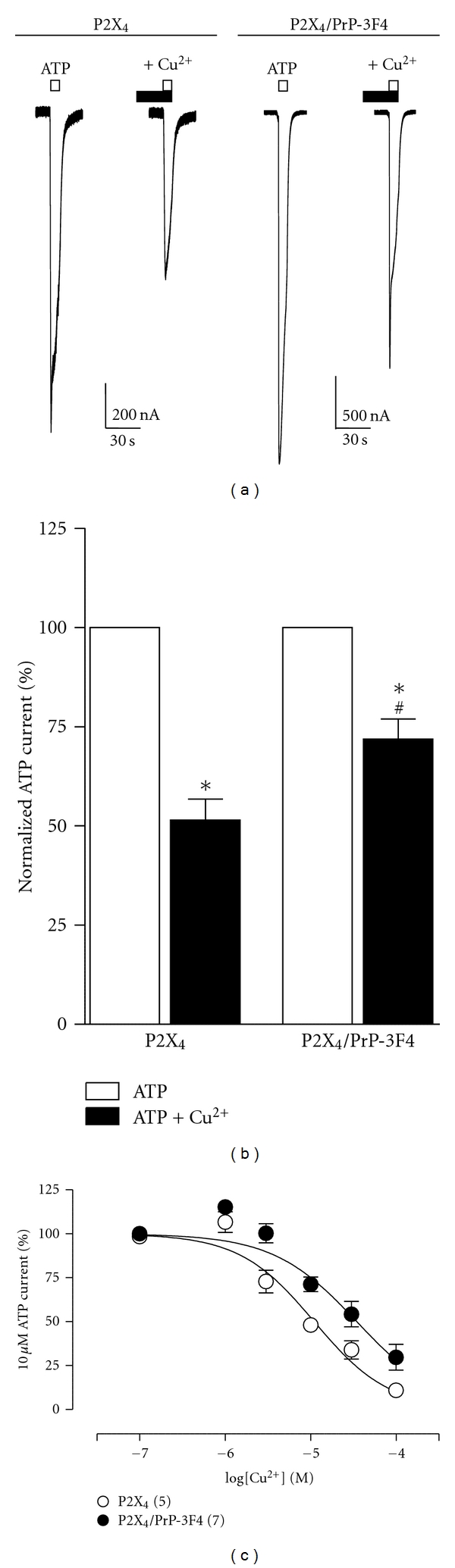
PrP^C^ prevents Cu^2+^-induced inhibition of P2X_4_ receptor. (a) Representative recordings obtained from oocytes expressing P2X_4_ receptor (left traces, P2X_4_) or coexpressing P2X_4_ receptor and PrP-3F4 (right traces, P2X_4_/PrP-3F4) showing 10 *μ*M ATP-evoked currents (*open bars*) and its inhibition by 10 *μ*M Cu^2+^ (*closed bars*). (b) Statistical analysis of Cu^2+^ inhibition showed in (a), performed in different oocytes (*n* = 12–14, *P < 0.01 versus ATP, ^#^ *P* < 0.01 versus P2X_4_ alone). Bars are mean values ± SEM. (c), Cu^2+^ concentration-response curves of 10 *μ*M ATP inhibition in oocytes expressing P2X_4_ receptor (open circles) or co-expressing P2X_4_ receptor and PrP-3F4 (closed circles). Symbols are mean values ± SEM, numbers in parenthesis are number of oocytes.

We assess the Cu^2+^-induced inhibition of 10 *μ*M ATP currents in oocytes expressing P2X_4_ receptors. The magnitude of the inhibition by 10 *μ*M Cu^2+^, preapplied during 30 s, was 51.5 ± 5.3% of the 10 *μ*M ATP-evoked currents (*n* = 14, Figures 3(a) and 3(b)). However, the 10 *μ*M Cu^2+^-induced inhibition was reduced only to 71.9 ± 5% of the 10 *μ*M ATP-evoked currents in oocytes co-expressing P2X_4_ receptors and the PrP-3F4 (*n* = 12, *P* < 0.05 compared to P2X_4_ alone, Figures 3(a) and 3(b)), showing that PrP-3F4 prevented the Cu^2+^-induced inhibition of P2X_4_ receptors compared to the Cu^2+^ inhibition elicited in oocytes expressing only this receptor. Furthermore, the presence of PrP-3F4 in the oocytes caused a rightward displacement of the Cu^2+^ concentration-response curve obtained in oocytes expressing only P2X_4_ receptor, an IC_50_ of 11.5 ± 1.9 *μ*M was obtained for P2X_4_ and 34.1 ± 7.6 *μ*M for P2X_4_/PrP-3F4 (*n* = 5–7, *P* < 0.01, Figure 3(c)), confirming that PrP-3F4 prevented the Cu^2+^-induced inhibition not only at low micromolar concentrations of Cu^2+^, but even at higher physiological concentrations of the metal.

## 4. Discussion

Several functions have been attributed to PrP^C^, including immunoregulation, signal transduction, copper binding, neurite outgrowth, induction of apoptosis or prevention of apoptosis against apoptotic stimuli, and others [41]. In addition, PrP^C^ has been related to synapse formation and maintenance and synaptic transmission [9, 10, 42], although the mechanisms by which it exerts its role is still unknown. One of the proposed targets for PrP^C^ in synapse is to modulate Cu^2+^ homeostasis, based on a highly conserved Cu^2+^-binding sequence located on its N-terminal domain, which includes four identical repeats of the peptide sequence Pro-His-Gly-Gly-Gly-Trp-Gly-Gln [12, 15, 16]. It is known that PrP^C^ binds Cu^2+^ with high affinity [14–17], and the octarepeat region of the human PrP^C^ (PrP_59-91_) reduces Cu(II) to Cu(I) *in vitro*, which depends on the tryptophan residues present in the octapeptide repeats [19, 20]. Cu^2+^ modulates synaptic transmission at micromolar concentrations by a wide range of mechanisms, be one of the most relevanting modulations of neurotransmitter receptors within glutamatergic, gabaergic, and purinergic synapses, among others [43, 44]. In a previous study, we demonstrated that Cu^2+^ at micromolar concentrations inhibits the ATP-evoked currents of P2X_4_ receptors [37]. Here we show that the full-length prion protein-expressed in *Xenopus* oocytes localizes in the cell surface and modulates the Cu^2+^ interaction with P2X_4_ receptor; oocytes which coexpressed PrP-3F4 and P2X_4_ receptors have a diminished Cu^2+^-induced inhibition of the ATP-evoked currents compared with oocytes which only expressed the P2X_4_ receptor. This reduced inhibition by Cu^2+^ was observed on Cu^2+^ concentration-response curves, where the IC_50_ of Cu^2+^ was significantly increased in the presence of PrP-3F4, indicating that PrP-3F4 can exert its modulatory role even at high micromolar concentrations of Cu^2+^, reached in the synaptic cleft after depolarization [45]. These results, together with our previous findings showing that coapplication of Cu^2+^ with the N-terminal PrP fragment (PrP_59-91_) prevents the inhibitory effect of copper on P2X_4_ receptors and even reverts the established Cu^2+^-induced inhibition of the P2X_4_ receptors [38], strongly support the idea that PrP^C^ could modulate synaptic copper and therefore affect the function of P2X_4_ receptors and synaptic transmission. 

In addition to the potential synaptic role of PrP^C^ driven by its ability to bind Cu^2+^, a known modulator of neuronal excitability [43, 44], there is increasing evidence of direct interaction between PrP^C^ and neurotransmitter receptors. PrP^C^ directly interacts with the NR2D subunit of the NMDA receptor, inhibiting it and preventing NMDA-induced excitoxicity in the hippocampus [46]. On the other hand, PrP^C^ also exerts a neuroprotective role against kainate-induced neurotoxicity in the hippocampus, probably by regulating differentially the expression of GluR6 and GluR7 kainate receptor subunits [47]. Moreover, PrP^C^ can modulate the activity of serotoninergic receptors signaling pathways in 1C11^5-HT^  cells [48]. We observed a slight, although not significant, reduction on ATP affinity of P2X_4_ receptor in the presence of PrP-3F4, this might suggest an interference with ATP binding or stabilization of closed states, although further experiments are required to evaluate this hypothesis. Altogether, these studies and the presented here highlight the modulatory role of PrP^C^ at synaptic transmission in CNS, involving direct regulation of neurotransmitter receptors and/or their signaling cascade, or indirectly, by controlling the synaptic levels of Cu^2+^.

The understanding of the physiological function of PrP^C^ on synaptic transmission may clarify the pathogenic processes underlying prion diseases. Based on our results, it is possible to suggest that the resulting cognitive deterioration of prion diseases could involve a loss of the modulatory role of PrP^C^ on brain function, as it is converted to the pathogenic isoform.

## Abbreviations

PrP^C^: Cellular prion protein
ATP: Adenosine triphosphate
CNS: Central nervous system
EC_50_: Median effective concentration
IC_50_: Median inhibitory concentration.
